# Molecular Dynamics Simulation of Hydrogels Based on Phosphorylcholine-Containing Copolymers for Soft Contact Lens Applications

**DOI:** 10.3390/molecules28186562

**Published:** 2023-09-11

**Authors:** Katarzyna Filipecka-Szymczyk, Malgorzata Makowska-Janusik, Wojciech Marczak

**Affiliations:** Faculty of Science and Technology, Jan Dlugosz University, Al. Armii Krajowej 13/15, 42-200 Częstochowa, Poland; k.filipecka-szymczyk@ujd.edu.pl (K.F.-S.); m.makowska@ujd.edu.pl (M.M.-J.)

**Keywords:** hydrogel, computer simulations, polymers, diffusion, free volume, hopping mechanism

## Abstract

The structure and dynamics of copolymers of 2-hydroxyethyl methacrylate (HEMA) with 2-methacryloyloxyethyl phosphorylcholine (MPC) were studied by molecular dynamics simulations. In total, 20 systems were analyzed. They differed in numerical fractions of the MPC in the copolymer chain, equal to 0.26 and 0.74, in the sequence of mers, block and random, and the water content, from 0 to 60% by mass. HEMA side chains proved relatively rigid and stable in all considered configurations. MPC side chains, in contrast, were mobile and flexible. Water substantially influenced their dynamics. The copolymer swelling caused by water resulted in diffusion channels, pronounced in highly hydrated systems. Water in the hydrates existed in two states: those that bond to the polymer chain and the free one; the latter was similar to bulk water but with a lower self-diffusion coefficient. The results proved that molecular dynamics simulations could facilitate the preliminary selection of the polymer materials for specific purposes before their synthesis.

## 1. Introduction

Hydrogels are hydrophilic three-dimensional networks of polymers chemically cross-linked and physically entangled with excellent swelling capacity in water [1]. Because their properties resemble those of body tissues, hydrogels are suitable for medical and pharmaceutical applications [2,3,4,5,6,7]. A breakthrough in ophthalmology occurred in the 1960s when Wichterle and Lim developed poly(2-hydroxyethyl methacrylate), P-HEMA, as a biocompatible synthetic hydrogel [8,9] for soft contact lenses (SCLs). Sixty years later, P-HEMA-based SCLs still are manufactured on a mass scale [10].

**Figure 1:**
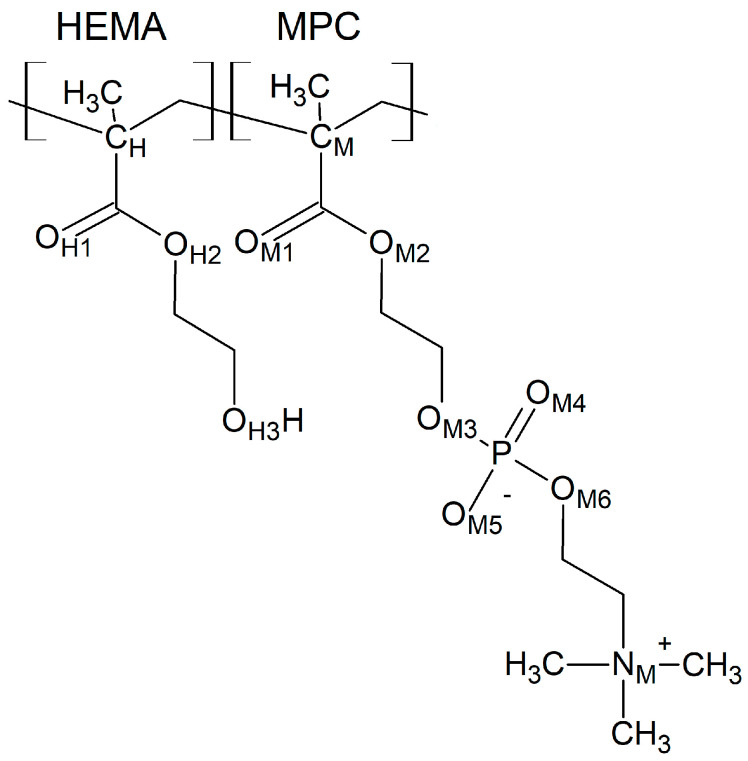
A fragment of the P(MPC–*co*–HEMA) copolymer chain. The atoms are tagged to simplify referring.

Hydroxyl and carbonyl functional groups in HEMA molecules (Figure 1) can hydrogen-bond water molecules. On the other hand, hydrophobic methyl groups in the polymer matrix ensure hydrolytic stability and mechanical strength. Moreover, P-HEMA is inert to many chemicals, resistant to degradation, thermally stable, and has good mechanical properties as an SCL material due to cross-linking of the chains [11,12,13,14,15]. The main disadvantage of pure P-HEMA hydrogels in ophthalmologic applications is low equilibrium water content (EWC) of only 38% by mass. This results in poor oxygen permeability as oxygen is transported mainly through the water phase of the hydrogels [16]. Consequently, long-term hypoxia and hypercapnia (increased level of carbon dioxide) may occur with the use of P-HEMA SCLs and lead to deterioration of health [17]. Copolymers of HEMA with compounds that bind more water are better in this respect [18,19,20].

High water-capacity units in the copolymer can be those of 2-methacryloyloxyethyl phosphorylcholine, MPC (Figure 1). The phosphorylcholine (PC) moiety enhances bio-inertia as a consequence of impaired electrostatic interactions and the formation of a hydration shell with a large amount of highly mobile free water around [21,22,23,24,25,26]. The latter strongly repulses the surrounding proteins [27,28]. Moreover, the PC moiety inhibits cell adhesion and reduces blood coagulation, and improves immune response [27,29,30]. These properties make MPC an ideal component of SCL biomaterials [31,32,33,34,35]. However, the properties of copolymers consisting of the same constituent units may vary depending on their arrangement in the chain. The chain arrangement may be complex even for linear biopolymers, which could be classified as statistical, random, or alternating. In the statistical arrangement, the sequential distribution obeys statistical laws, e.g., Markovian statistics, while in the random distribution, the probability of a given mer occupying a site in the chain does not depend on the adjacent mers. Block copolymers are composed of block macromolecules built of many constitutional units each [36].

The polymer chain arrangement has a significant influence on the physicochemical properties of the materials but it is difficult to test experimentally. On the other side, despite significant progress in improving the properties of the materials used in the production of SCLs, searching for new biomaterials for advanced ophthalmological applications is still a matter of interest. Computer simulations, complementary to experimental studies, predicting the physicochemical properties of polymers may substantially shorten the time necessary for designing new polymer materials and give a new look at their properties. Nevertheless, reports on computer simulations of the physicochemical properties of polymers potentially applicable in ophthalmology are scarce [37,38,39,40,41,42,43,44,45,46,47,48,49,50,51,52]. Therefore, the present work was aimed at filling this gap at least partially. The attention was focused on the structural and dynamic properties of the P(MPC–*co*–HEMA) hydrogels, especially on the hydration phenomena in these materials. We studied the mechanism of water diffusion which influences the transport of oxygen through the hydrogels. Proper oxygen concentration on the eyeball surface is crucial for the health and comfort of the SCL wearers. Understanding transport processes in polymeric materials facilitates an assessment of their suitability for ophthalmological applications.

## 2. Results and Discussion

In the simulations, we considered several intermolecular distances and interactions. To keep the text readable, we used shortcuts rather than full descriptions of the atoms and interactions. Thus, we marked the atoms by the subscripts H, M, and W, which denote HEMA, MPC, and water molecules, respectively. The full identification of the symbols for HEMA and MPC is given in Figure 1.

Copolymers of 2-hydroxyethyl methacrylate (HEMA) with 2-methacryloyloxyethyl phosphorylcholine (MPC), P(MPC–*co*–HEMA), dry and hydrated with water content 10, 20, 40, and 60% by mass were investigated. The P(MPC–*co*–HEMA) copolymer chains in the simulations were composed of 50 mers, 13 of one kind and 37 of the other, in block or random configuration. The following four copolymers were studied:

- block M13H37 (B13),
- block M37H13 (B37),
- random with 13 MPC and 37 HEMA mers,
  HMH6MH4MH4MH4MHMHMHMH11MHM2H2M2H (R13),
- random with 37 MPC and 13 HEMA mers,
  M10HM4H2M6H3M5H4MHM5HM3HM3 (R37),

where M stands for MPC and H for HEMA, the subscripts denote the number of mers, and codes in parentheses identify the copolymer. From now on, the codes will be used for simplicity.

### 2.1. Structure and Chain Packing

The flexibility of the HEMA and MPC mers would be manifested in differences in their geometry resulting from the copolymer chain arrangement and degree of hydration. We chose distances between two atoms in each of the mers as indicators of this property. For both mers, the first atom was that of carbon belonging to the copolymer main chain, i.e., C_H_ or C_M_ in Figure 1. The second atom was located at the end of the side chain: the O_H3_ of HEMA and N_M_ of MPC.

**Figure 2:**
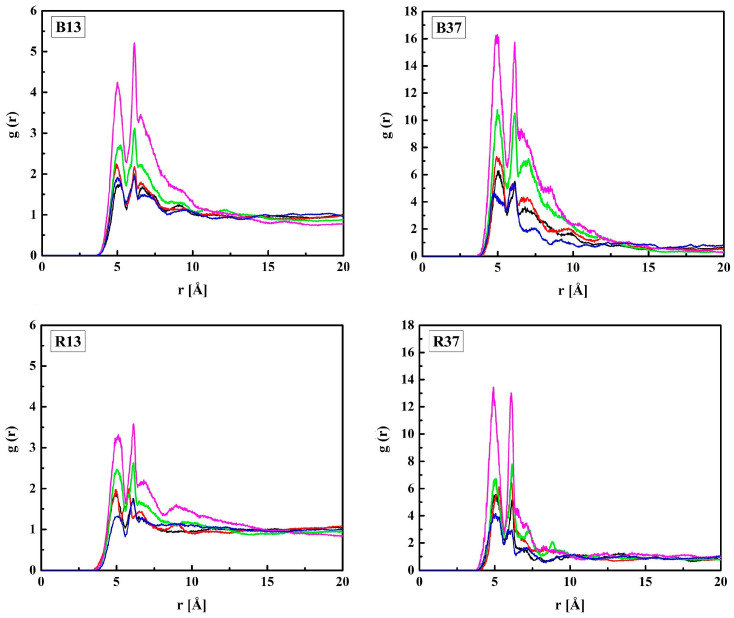
Radial distribution function *g*(*r*) of the C_H_–O_H3_ pair of HEMA in the B13, B37, R13, and R37 copolymers. Blue line—non-hydrated polymer; black, red, green, and magenta lines—hydrated polymers containing 10, 20, 40, and 60% of water by mass, respectively.

First, the HEMA chains will be discussed. The radial distribution functions *g*(*r*) of the C_H_–O_H3_ pairs are shown in Figure 2. The shortest distance between C_H_ and O_H3_ atoms assessed from the RDF first peak maximum is 5.0 Å for all the systems studied. Thus, neither the arrangement of the copolymer chain nor the hydration affects the HEMA geometry. This proves that the HEMA mer is relatively rigid. Higher *g*(*r*) peaks for hydrated polymers result from the lower number density of HEMA mers in cells enlarged for accommodating water molecules (cf. Table 1).

**Figure 3:**
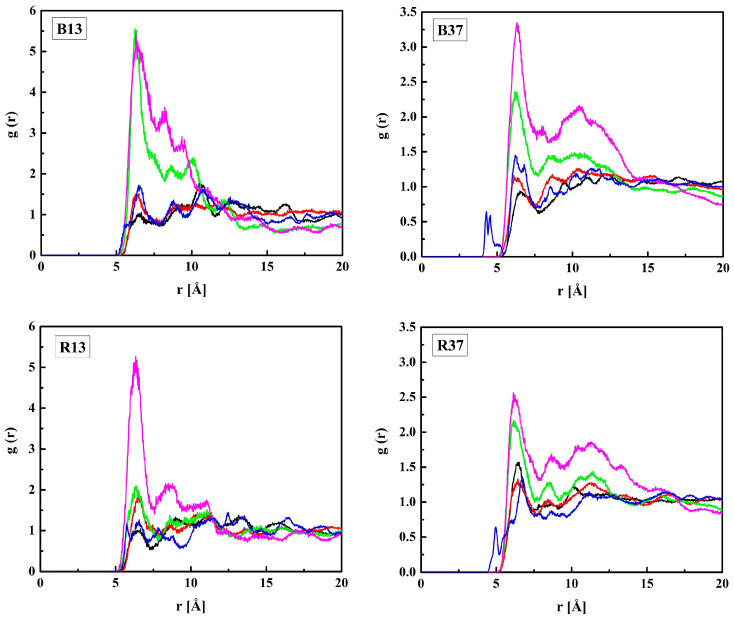
Radial distribution functions *g*(*r*) of the C_M_–N_M_ pair of MPC in the B13, B37, R13, and R37 copolymers. Blue line—non-hydrated polymer; black, red, green, and magenta lines—hydrated polymers containing 10, 20, 40, and 60% of water by mass, respectively.

**Figure 4:**
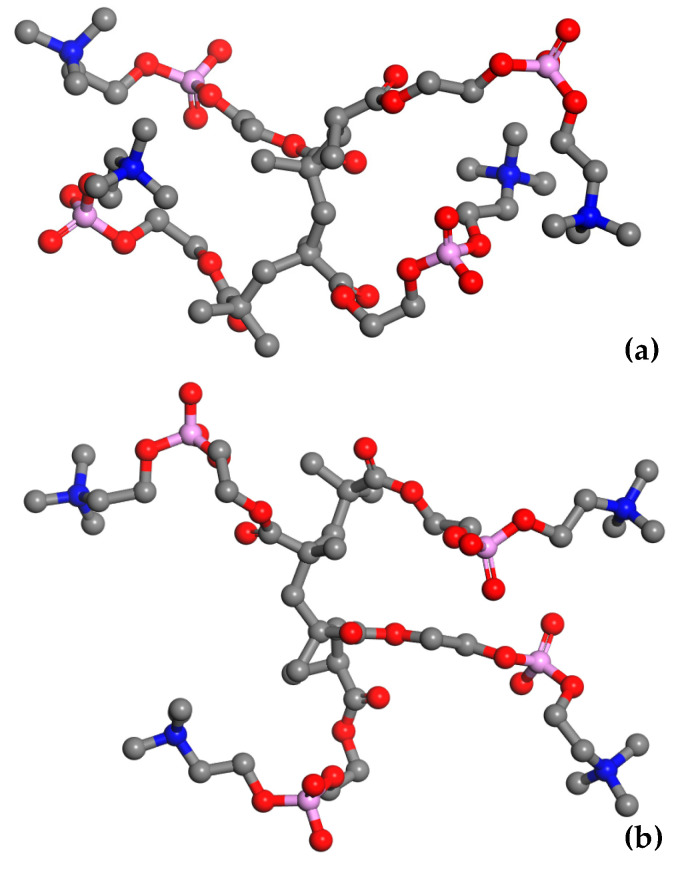
Snapshots of the simulated adjacent MPC side chains in non-hydrated block copolymer (**a**) and that containing 60% of water by mass (**b**). Hydrogen atoms and water molecules were omitted for the picture clarity. Atoms: C—grey, O—red, N—blue, and P—magenta.

The MPC groups are different in this respect. Figure 3 shows the RDFs calculated for the C_M_–N_M_ pair. For non-hydrated copolymers, the C_M_–N_M_ interatomic distance is equal to 5.7 Å for R13 and 5.6 Å for B13. It is significantly shorter for the copolymers with 37 mers of MPC in the chain: 4.9 Å and 4.3 Å for R37 and B37, respectively. Thus, the MPC chains are stiffened by those of HEMA in their vicinity, while they can coil when in bigger blocks formed by MPC mers. The C_M_–N_M_ distances shorter than the C_H_–O_H3_ ones seem rather odd because the MPC mer is almost twice as long as that of HEMA. An explanation could be the coulombic attraction between oppositely charged trimethylammonium and phosphate groups of adjacent mers causing twisting and shortening of the side chain of the polymer (Figure 4).

The C_M_–N_M_ distance increases due to the hydration of the polymer, for R37 and B37 in particular, and eventually reaches 6.3 Å for the four systems with 60% of water. However, the MPC chains remain curved. For comparison, the distance in a fully straightened chain would be about 11.2 Å, as estimated from the molecule geometry and bond lengths. The elongation of C_M_–N_M_ distance suggests that water penetrates the voids between the MPC side chains and influences their shape. Two mechanisms could lead to this effect. First, non-specific hydration of the trimethylammonium and phosphate groups may weaken the coulombic attraction between the adjacent chains because of the high dielectric constant of water. Second, water bridges hydrogen-bonded to the chains may stabilize their geometry. This will be discussed in the next sections.

Although some structural features are manifested in the reported RDFs, the lack of long-range regularity evidences the amorphousness of the studied polymers.

### 2.2. Distribution of Water Molecules

**Figure 5:**
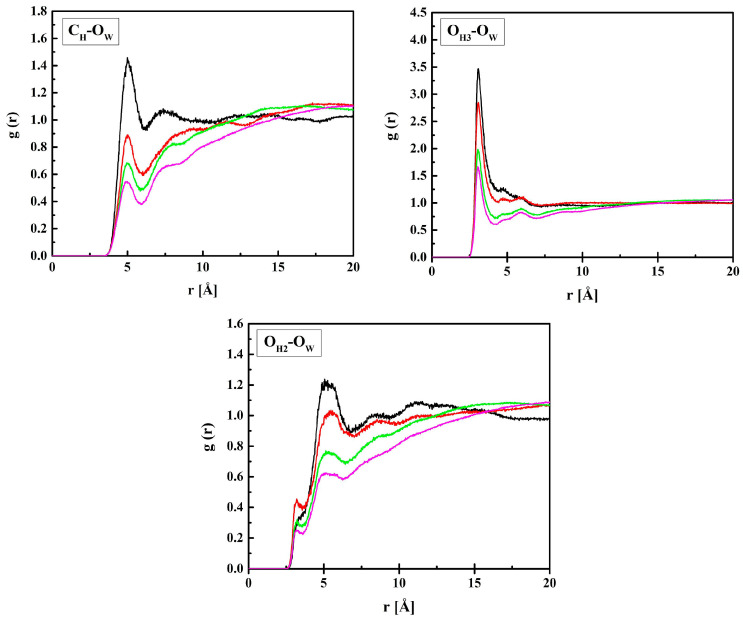
Radial distribution functions *g*(*r*) for atom pairs: water oxygen (O_W_) and C_H_, O_H3_, and O_H2_ of HEMA for the R37 copolymer with various water content. Black, red, green, and magenta lines—hydrated polymers containing 10, 20, 40, and 60% of water by mass, respectively.

In this section, the location of water molecules in the hydrogels will be discussed in detail beginning from the vicinity of HEMA chains. The RDF functions of the C_H_–O_W_, O_H3_–O_W_, and O_H2_–O_W_ pairs calculated for the hydrated R37 copolymer are plotted in Figure 5.

The RDF functions for B13, B37, and R13 are similar to the presented ones. The shortest distance between O_H3_ and O_W_ is 3.1 Å and it does not depend on the amount of water in the system, at least within the concentration range studied. Similarly, a short distance equal to 3.3 Å is between O_H2_ and O_W_, but the respective *g*(*r*) peak is tiny, while a pronounced maximum occurs at 5.2 Å. The two ca. 3 Å-long distances suggest hydrogen bonds occurring between water molecules and the ether or terminal hydroxyl groups of the HEMA chain. The second interaction predominates over the first most probably because of the main polymer chain hindering the access of the water OH group to the carbonyl oxygen atom. The O_H2_–O_W_ distance of 5.2 Å, corresponding to the pronounced peak at *g*(*r*), and that of 5.0 Å between C_H_ and O_W_ are typical of non-hydrogen-bonded molecules.

**Figure 6:**
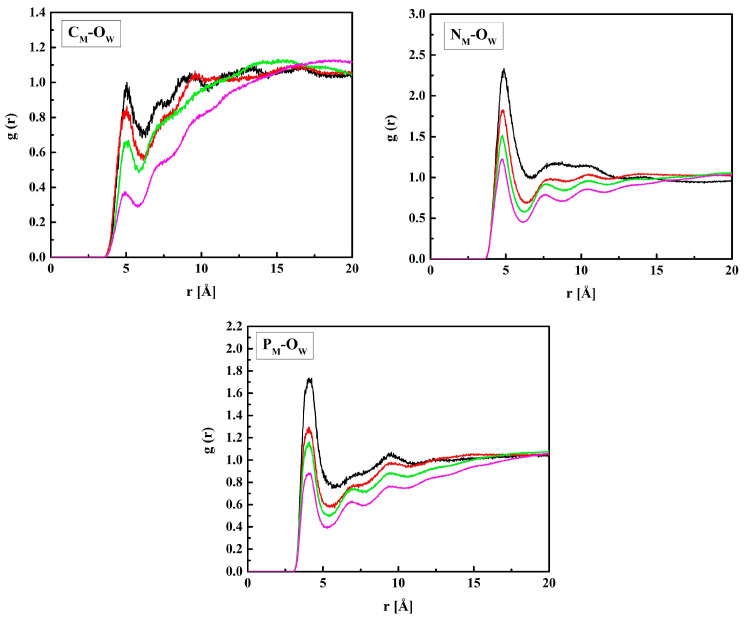
Radial distribution functions *g*(*r*) for atom pairs: water oxygen (O_w_) and C_M_, N_M_, and P_M_ of MPC for the R37 copolymer with various water content. Black, red, green, and magenta lines—hydrated polymers containing 10, 20, 40, and 60% of water by mass, respectively.

Figure 6 shows the RDF functions for MPC + water: C_M_–O_W_, N_M_–O_W,_ and P_M_–O_W_ pairs. The shortest distance between P_M_ and O_W_, 4.1 Å, suggests that water molecules gather in the vicinity of the negatively charged phosphate group. Similar gathering around the positive trimethylammonium group also occurs, but at a longer distance of 4.7 Å because of the three blocking methyl groups. The distance between C_M_ and O_W_, 5.0 Å, is equal to that between C_H_ and O_W_. Indeed, the C_H_ and C_M_ atoms belong to the main polymer chain, and the HEMA and MPC side chains are identical up to the second O atom (O_H3_ and O_M3_).

### 2.3. Hydrogen Bonding

To evaluate the feasibility of water–polymer hydrogen bonds, we considered all potential hydrogen-bonding sites: the oxygen, nitrogen, and phosphorus atoms in HEMA and MPC mers, as well as hydrogen atoms from water molecules. As in previous sections, we will begin the discussion from the HEMA mers.

**Figure 7:**
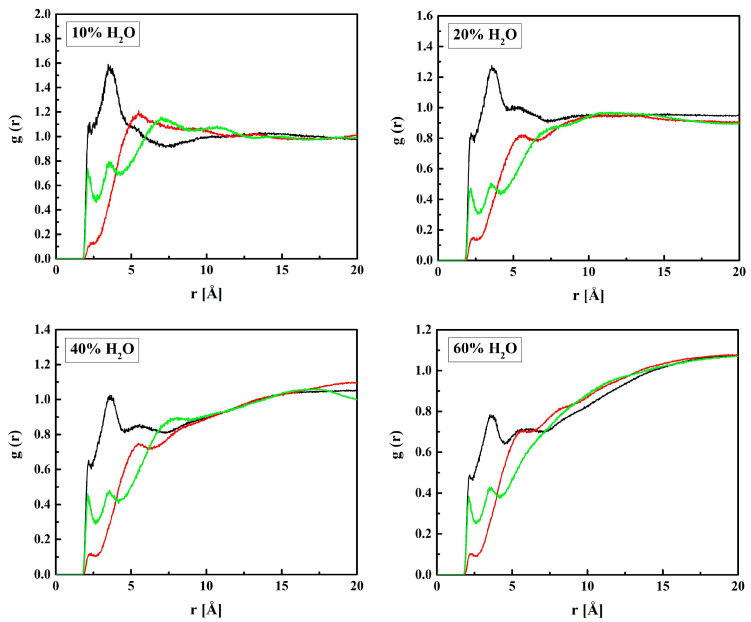
Radial distribution functions *g*(*r*) for atom pairs: water hydrogen and HEMA oxygen for the R37 copolymer with various water content. Green lines: O_H1_–H_W_, red: O_H2_–H_W_, and black: O_H3_–H_W_.

**Figure 8:**
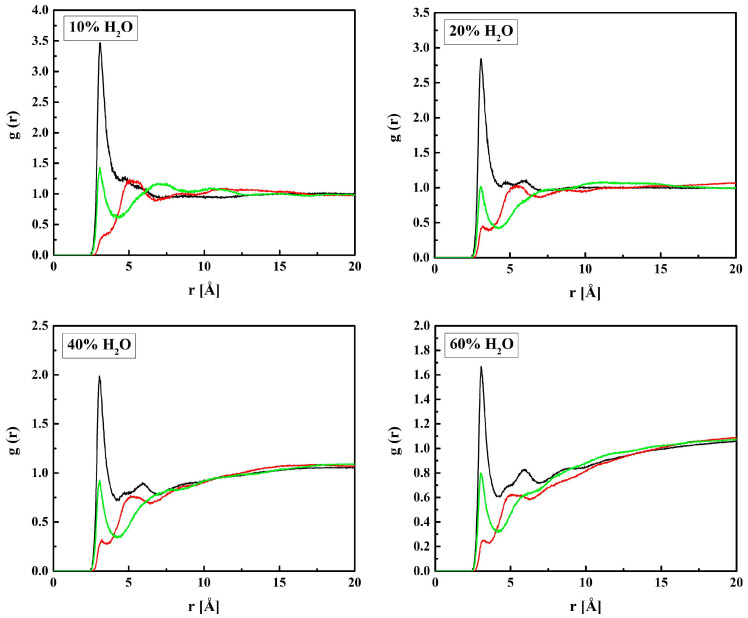
Radial distribution functions *g*(*r*) for atom pairs: water oxygen and HEMA oxygen for the R37 copolymer with various water content. Green lines: O_H1_–O_W_, red: O_H2_–O_W_, and black: O_H3_–O_W_.

The RDF functions for the three pairs (O_H1_ or O_H2_ or O_H3_ in R37)–H_W_ are shown in Figure 7. The distances O_H1_–H_W_ and O_H3_–H_W_ corresponding to the RDFs’ first maxima are equal to 2.2 Å suggesting hydrogen bonds. However, the hydroxyl groups (O_H3_) are probably donors of the hydrogen atoms rather than acceptors. That could be concluded because the first peak of the respective RDF is just a small local maximum preceding a steep increase up to the global maximum at *r* ≈ 3 Å for the systems with water content not exceeding 40% by mass. The O_H3_–H…O_W_ bonds seem very probable because of the short O_H3_–O_W_ distance equal to 3.1 Å (see Figure 8). Contrary to O_H3_, the ether and carbonyl oxygen atoms, O_H2_ and O_H1_, can only be acceptors in hydrogen bonding. From these two, O_H1_ is doubtless the stronger acceptor that is manifested in prominent first maxima at *r* = 3.1 Å and the long-range oscillations of the O_H1_–O_W_ RDFs. The oscillations would be reinforced by the cooperativity of hydrogen bonds. However, the cooperativity could not be detected because the electron structures of the modeled molecules remained unchanged during the simulation. Of course, increased water concentration causes more water molecules to stay away from the oxygen atoms of HEMA and the *g*(*r*) maxima to decrease. That has been reported for other hydrated polymers as well [53,54].

**Figure 9:**
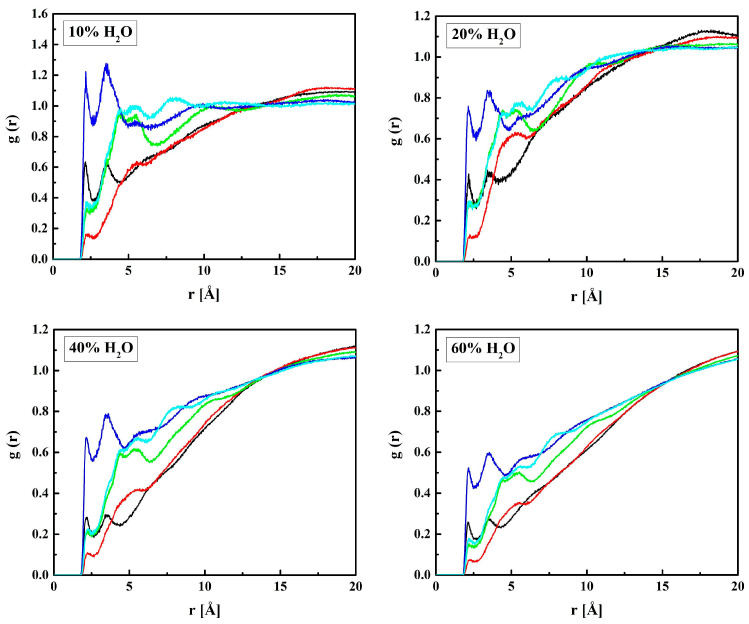
Radial distribution functions *g*(*r*) for atom pairs: water hydrogen and MPC oxygen for the R37 copolymer with various water content. Black lines: O_M1_–H_W_, red: O_M2_–H_W_; green: O_M3_–H_W_, blue: O_M(4,5)_–H_W_, cyan: O_M6_–H_W_.

**Figure 10:**
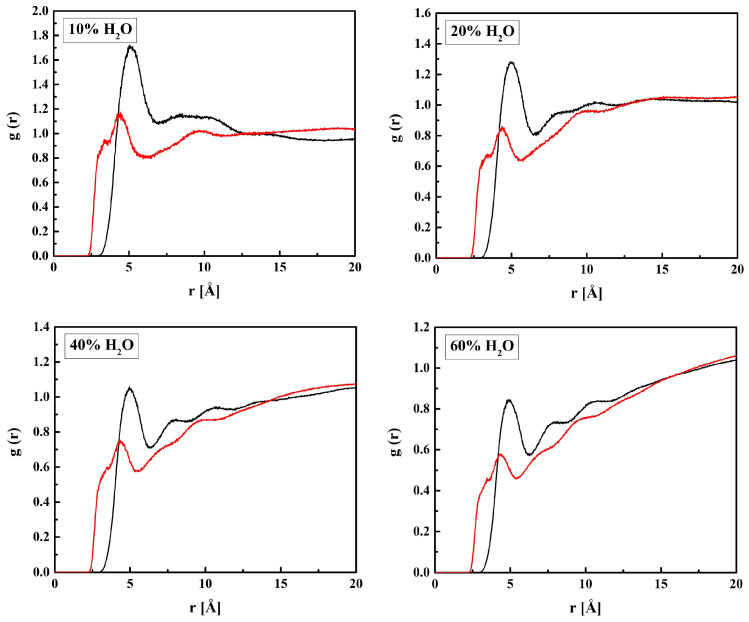
Radial distribution functions *g*(*r*) for atom pairs: water hydrogen and MPC nitrogen or phosphorus for the R37 copolymer with various water content. Black lines: N_M_–H_W_, red: P_M_–H_W_.

**Figure 11:**
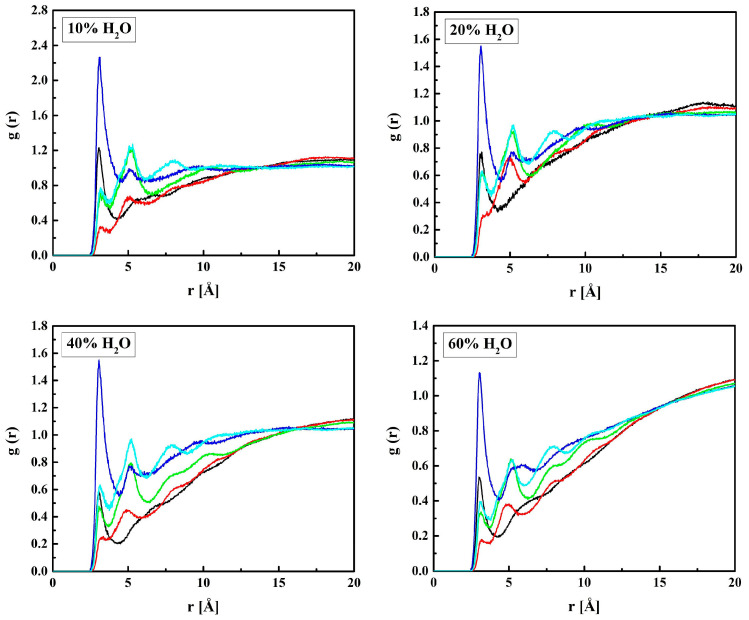
Radial distribution functions *g*(*r*) for atom pairs: water oxygen and MPC oxygen for the R37 copolymer with various water content. Black lines: O_M1_–O_W_, red: O_M2_–O_W_; green: O_M3_–O_W_, blue: O_M(4,5)_–O_W_, cyan: O_M6_–O_W_.

**Figure 12:**
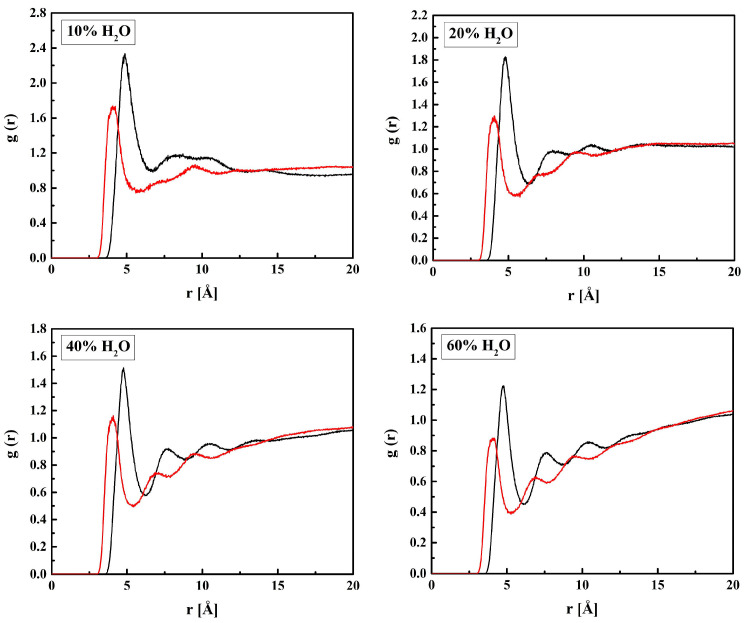
Radial distribution functions *g*(*r*) for atom pairs: water oxygen and MPC nitrogen or phosphorus for the R37 copolymer with various water content. Black lines: N_M_–O_W_, red: P_M_–O_W_.

MPC is a derivative of HEMA with phosphocholine substituted for hydrogen in the terminal hydroxyl group, cf. Figure 1. Thus, the investigation of the MPC hydration shell was focused on two questions: (i) How are water molecules located around the phosphocholine group, and (ii) Is the arrangement in the remaining area different from that around HEMA? Only the results for R37 are reported. Those for the three other copolymers are similar. The RDFs for six atom pairs, O_M1,…, M6_–H_W_, as well as N_M_–Hw and P_M_–H_W_, are plotted in Figure 9 and Figure 10. Respective functions for the pairs with O_W_ rather than H_W_ are reported in Figure 11 and Figure 12. The RDFs for the carbonyl and ether oxygen atoms in MPC, O_M1_, and O_M2_, do not differ significantly from the respective functions obtained for HEMA. This suggests that hydration spheres around these fragments of the two sidechains are similar to one another.

Answering the second question requires a more detailed discussion. The RDFs for O_M4_ and O_M5_ are pretty much the same because the two atoms are equivalent in an MPC molecule, as illustrated by the resonance structures:


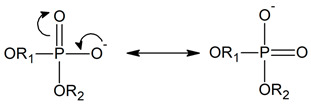


Sharp peaks on the RDFs, these (see Figure 9) and those for P_M_-O_W_ (see Figure 12) and P_M_-H_W_ (see Figure 10), confirm a high affinity of water to the phosphate group. Thus, water forms hydrogen bonds with the O_M4_ and O_M5_ atoms that result in the RDF first peak coordinates of 3.1 Å and 2.2 Å for the O_M(4,5)_–O_W_ and O_M(4,5)_–H_W_ pairs, respectively. The first peak coordinates for O_M3_ and O_M6_—water pairs are equal to 3.1 Å and 2.2 Å too, but the peaks are significantly lower. This suggests the formation of hydrogen bonds involving the O_M4_ and O_M5_ atoms facilitated by the negative charge of the phosphate group attracting water molecules through the ion–dipole interaction. We should keep in mind that hydrogen bonding is a purely electrostatic interaction in this simulation. For O_M3_ and O_M6_, the effect is weaker if any.

The affinity of water to the nitrogen atom of the choline group, N_M_, is strong, as the first peaks on the RDFs for N_M_–O_W_ (see Figure 12) and N_M_–H_W_ (see Figure 10) are high and sharp. However, the hydrogen bonding between N_M_ and water does not occur because of the steric hindrance by the three methyl groups. Thus, the ion–dipole attraction is the main force that keeps water molecules in the vicinity of the group. Doubtless, the preferred orientation of a water molecule is that with the pairs of lone electrons directed towards the positively charged nitrogen atom. It is manifested in the RDFs maxima at 4.7 Å for N_M_–O_W_ and at 5.0 Å for N_M_–H_W_.

### 2.4. Free Volumes

**Figure 13:**
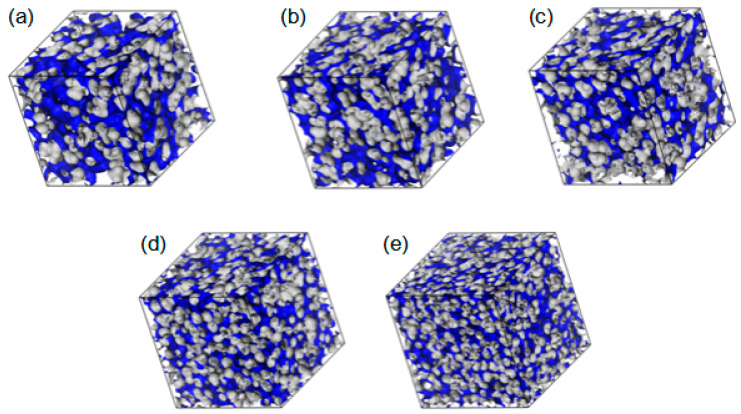
Morphology of free volumes in R37, dry (**a**) and with different water content: 10% (**b**), 20% (**c**), 40% (**d**), and 60% (**e**) by mass for the probe of infinitesimal radius. The walls of the empty channels making up the free volumes are marked in blue.

The fractional free volumes (FFV) for twenty P(MPC–*co*–HEMA) systems determined for a probe with a radius of 0 Å (cf. Section 3.2) are reported in Table 2. The considered polymer material consisted of the copolymer itself and, if it was hydrated, water as well. Figure 13 shows an illustration for R37. Morphologies for the other polymer systems are similar to these, albeit the FFVs for R37 and B37 are slightly bigger than those for R13 and B13 at low water contents in particular. The sequence of mers (block or random) does not affect the FFV significantly. The FFVs decrease with the increase in water content (Table 2), evidencing that swelling does not compensate for the filling of the voids by water. Moreover, the average void shrinks when the water content increases.

**Figure 14:**
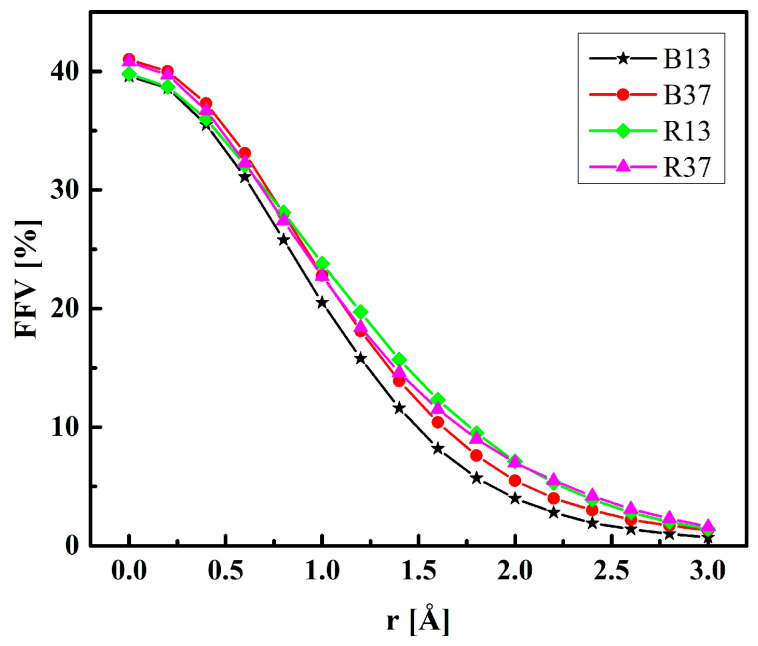
The fractional free volume (FFV) of non-hydrated P(MPC–*co*–HEMA) as determined with probes of various radii.

In real systems, the free volumes for an infinitesimal probe are probably less important than those for molecules of finite size. Figure 14 shows the FFV of non-hydrated P(MPC–*co*–HEMA) copolymers tested with probes of various radii. The FFV is the smallest in B13 independently of the probe size. For the probes of the “molecular” size, i.e., those of radii above 1.5 Å, the sequence of polymers according to the FFVs is the following: B13 < B37 < R13 ≈ R37. This confirms the intuition—in random arrangements, the phosphocholine groups of long MPC side chains are separated one from another by broader spaces than in the block configurations. That makes penetration of the interchain spaces easier.

Another problem is an influence of swelling on the volume available for water in the polymers. To solve it, the calculations for a hard-sphere probe with a radius of 1.4 Å were performed. In these calculations, the polymers’ geometries remained as they were when swelled, while the hydration water was removed. The results are reported in Table 3. In dry copolymers, the FFVs available for water are bigger for random than block configuration. From among the four dry polymers, B13 shows the smallest fractional free volume of up to 20% of water by mass. However, the differences are small and vanish for higher water content.

**Figure 15:**
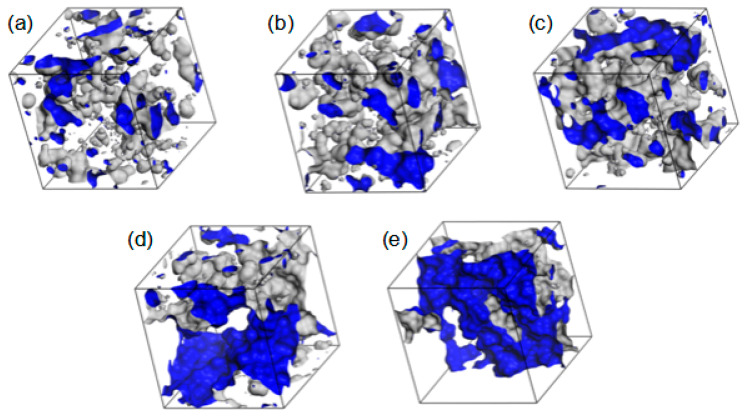
The volume in the simulated R37 material available for or filled with water: dry (**a**) and hydrated copolymers containing 10% (**b**), 20% (**c**), 40% (**d**), and 60% (**e**) of water by mass. The walls of polymer channels available for the probe with a radius of 1.4 Å (i.e., a “water molecule”) are marked in blue.

Examples of the free volume geometry in R37 are drawn in Figure 15. As the polymer hydration increases, the free volumes around the polymer chains become bigger. Eventually, pronounced diffusion channels arise. In the 40 and 60% hydrates, water is not only adsorbed into the polymer matrix but also accumulated around the rolled chains. Such “free” water would not interact directly with the polymer chains but resemble bulk water. This conclusion agrees with the experimental results evidencing bulk water in the hydrates containing at least 30% of water by mass [52,55,56].

### 2.5. Dynamics of the Polymer Chains

**Figure 16:**
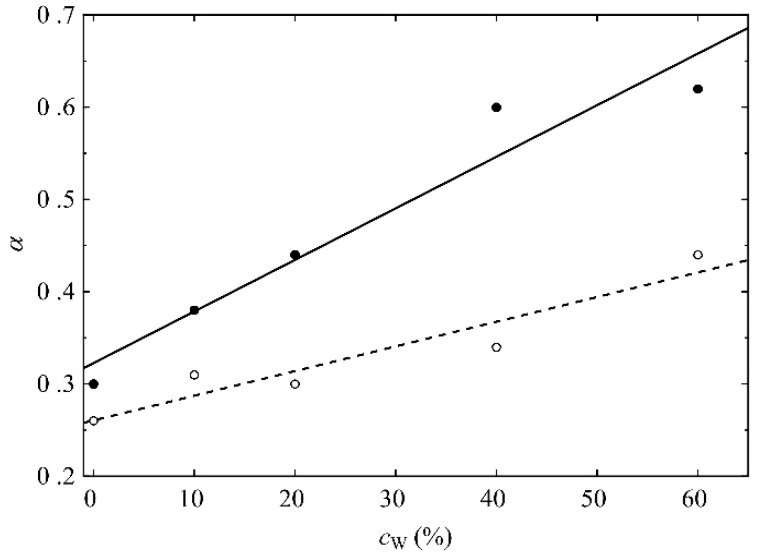
The exponents in the power law (Equation (1)) that evidence subdiffusion of the O_H3_ (open symbols) and N_M_ atoms (filled symbols) of R37 vs. the mass concentration of water. Lines are guides for the eye only.

The mean square displacement (MSD; cf. Equation (7)) is a measure of the polymer chains’ mobility. Thus, the MSDs were calculated from the MD-generated trajectories. Since the polymers are not typical fluids, the subdiffusion occurs rather than Brownian motions. Consequently, the exponent *α* in the power law:

(1) $$\langle r^{2}(t)\rangle =K_{\alpha }t^{\alpha }$$

is smaller than 1 rather than *α* = 1 typical of the diffusion described by the Einstein and Smoluchowski equation. The exponents *α* for hydroxyl O_H3_ and N_M_ atoms of R37 with different water content are plotted in Figure 16. They were calculated by the least squares fitting of the logarithmic form of Equation (1):

(2) $$\ln\langle r^{2}(t)\rangle =\ln K_{\alpha }+\alpha \ln t,$$

where *K_α_* is the generalized diffusion coefficient. The time interval for the fit was ca. 500 ns-long, starting from the thirtieth ps of the simulation.

The polymer chains meander around equilibrium points in the medium of complex structures where the movement of the molecules is impeded. Thus, the average time of jumping from one equilibrium point to another is infinite. The more mobile the molecules are, the higher *α* is in Equations (1) and (2). For each water concentration, the N_M_ atom of the MPC phosphorylcholine group is more mobile than the O_H3_ of the HEMA hydroxyl one. Like the previously analyzed RDF functions, this suggests high flexibility of the MPC side chains (cf. Figure 3 and Figure 4) that favors the formation of diffusion channels [57,58]. The side chains are more mobile in the hydrated polymers because water acts as a plasticizer. Small water molecules penetrate the space between chains that increases free volumes [59], cf. Figure 15. Consequently, the hydrated polymers would be less viscous.

Molecular models of Rouse [60] and Zimm [61] applied for polymers in semidilute and dilute solutions, respectively, provide insight into polymer dynamics. In the Rouse model, the polymer chain is represented by beads connected by springs. Each bead interacts with its neighbors through elastic forces and undergoes friction forces from the surroundings. Zimm extended Rouse’s approach by considering hydrodynamic interactions that alter the beads’ dynamics. A force applied to one chain segment distorts the velocity field in the rest of the fluid influencing the motion of other segments. The distortion slowly vanishes with increased distance. The MSD obeys the following scaling behavior [62]:

(3) $$\langle r^{2}(t)\rangle \propto \left\{\begin{array}{c} t^{1}\;for\;t<\tau_{m}\\ t^{\alpha }\;for\;\tau_{m}<t<\tau \\ t^{1}\;for\;t>\tau \end{array}\right.$$

where *τ*_m_ is the relaxation time of a monomer, and *τ* is the longest relaxation time of the chain. In the Rouse model, *α* = 0.5 for ideal chains and *α* = 0.54 for self-avoiding ones, while *α* = 0.67 in the Zimm model. Thus, the hydrodynamic interactions result in higher values of *α*.

The HEMA chain dynamics can be explained in terms of the Rouse model. In this case, there is no need to consider the hydrodynamic interactions in dry and hydrated polymers. MPC chains behave similarly in systems containing up to 20% water by mass independently of the simulation time and in those with 40 and 60% water until ca. 30 ps is reached. At longer simulation times, *α* ≥ 0.6 suggests hydrodynamic interactions considered in the Zimm model. Thus, the simulated polymers are subjected to reptational movements, and interactions between neighboring side chains hamper their motions. Only in highly hydrated polymers can the MPC side chains move more freely thanks to the surrounding water molecules.

### 2.6. Dynamics of Water Molecules

Exponents *α* in Equation (1) for the MSD of water molecules in hydrated polymers were calculated in the same manner as those for O_H3_ and N_M_ atoms. The only difference was the fitting range, which covered much longer time intervals exceeding 1000 ps. The values of exponents *α* reported in Table 4 suggest subdiffusion of water in hydrated copolymers at least up to the water content of 20% by mass, cf. Equation (3). In the systems containing 40 and 60% water, normal diffusion probably occurs as the exponents *α* are equal to 1 within the uncertainty limits of the fit at the confidence level of 95%. Higher exponents for the MPC-rich copolymers evidence that water is more mobile in these systems. The block or random arrangement of mers is not essential for mobility. These results are consistent with those discussed earlier. The hydroxyl and carbonyl groups of HEMA, and the carbonyl, choline, and phosphate groups of MPC form hydrogen bonds with surrounding water molecules and restrict their motions. In this manner, the so-called bound water arises. In highly hydrated systems (40 and 60% of H_2_O), water may resemble the bulk one to some extent. This is evidenced by the diffusion coefficients *D* (see Equation (8)) reported in Table 5. Their values amount from 53 to 58% of 2.84 × 10^−5^ cm^2^s^−1^ for bulk water at 307 K [63]. Thus, the decrease in mobility of water molecules caused by the polymer network is similar to that due to the cooling of water from 307 K to 285 K. In the latter temperature, *D*_H2O_ = 1.62 × 10^−5^ cm^2^s^−1^ [63]. Such a phenomenon was also reported by other researchers [37,64]. Although the diffusion coefficients are higher in copolymers with 37 MPC mers than in those with 13, the difference is small. Thus, flexible MPC chains can create efficient diffusion channels even in systems where they are outnumbered by HEMA mers. Moreover, water molecules in the outer layers of thick hydration shells of phosphorylcholine groups are fairly mobile [21,22,23,24,25,26].

**Figure 17:**
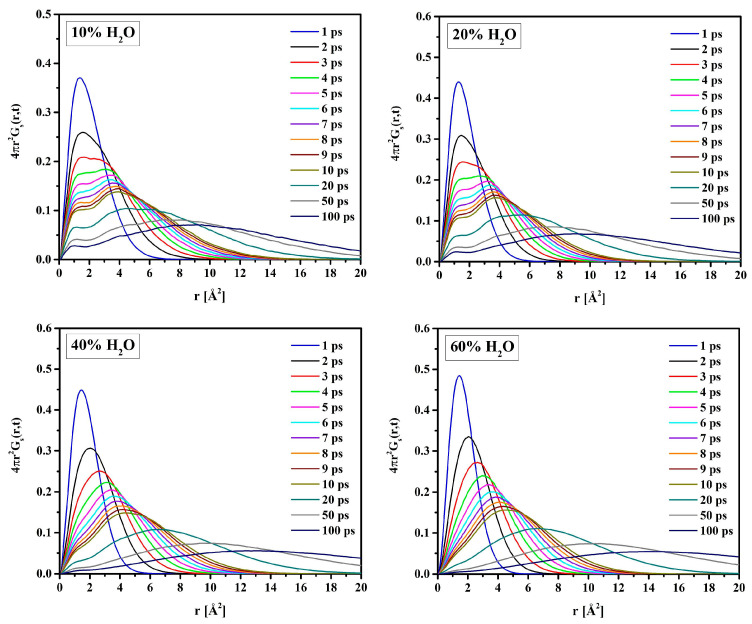
Van Hove self-correlation functions $G_{s}\left(\vec{r},t\right)$ calculated for water molecules in R37.

In the suggested two-state approach, the molecules’ mobility in the bound water differs from that in the bulk-like one. Analysis of the self-term of the van Hove space-time correlation functions (Equation (11)) supports this idea. Figure 17 shows $G_{s}\left(\vec{r},t\right)$ for oxygen atoms of water molecules (O_W_) in hydrated R37 in time intervals *t* from 1 to 100 ps long. For short simulation times, the distribution is unimodal. The single peak at *r* = 1.5 Å is due to vibrations of O_W_ within local “cages” formed by the neighboring atoms. Initially, all atoms vibrate in their original positions, and only after some time can jump to the adjacent cage. Therefore, multiple peaks, at least two, suggest a “hopping” of O_W_ from one cage to another for the longer simulation times. This is evident for the systems with 10 and 20% water. For more hydrated systems, the effect of “hopping” is masked by that of the self-diffusion of free water. Consequently, the self-correlation function is rather flat with a single maximum at *r* much bigger than the hydrogen bond length.

## 3. Computer Simulations

### 3.1. Model Construction and Simulation Details

Molecular dynamics (MD) simulations were performed with the Amorphous Cell and Forcite modules of the Materials Studio package [65]. To avoid calculation errors resulting from incorrect modeling of the starting structure, three modeled systems were constructed for each polymer configuration, and then the obtained results were averaged for each atomic system

In the first step, the geometries of four polymer chains were optimized with the Smart method implemented in the Forcite module. The DREIDING force field (FF) [66] suitable for polymer systems [50,51,67,68] was applied. The main reason for choosing the DREIDING FF was the intention to model hydrogen interactions and hydrogen bonds. The proposed FF describes the hydrogen interactions explicitly by Lennard-Jones 12–10 potential [66]. In this FF, the total potential energy *E*_total_ is the sum of the interaction energy terms:

(4) $$E_{total}=E_{bond}+E_{non-bond}=E_{B}+E_{A}+E_{T}+E_{I}+E_{vdW}+E_{Q}+E_{hb}.$$

The bond interaction energy *E*_bond_ is due to chemical bonding. It comprises terms resulting from the bond stretching and bending (*E*_B_), flat (*E*_A_), torsion (*E*_T_), and improper (*E*_I_) angle components. The non-bond term (*E*_non-bond_) encompasses van der Waals (*E*_vdW_) and electrostatic (*E*_Q_) interactions, and hydrogen bonds (*E*_hb_) [66].

The maximum number of geometry optimization cycles and the energy convergence criterion were set to 1000 and 0.001 kcal/mol, respectively. Each isothermal-isochoric (*NVT*) MD simulation was 5-ns-long with a time step of 1 fs. To check the sufficiency of the simulation time, the non-hydrated B13, B37, R13, and R37 systems were simulated for 10 ns. In all cases, the system was equilibrated thermodynamically after 3 ns. The total energy of the system was kept constant for simulations longer than 3 ns. All structures reached thermodynamic equilibrium during the simulation. The equations of motion were integrated using the Verlet algorithm [69]. The MD simulations were controlled by the Nosé-Hoover thermostat [70] set to a temperature of 307 K equal to the average for the eyeball surface [71]. Copolymer configurations obtained in this manner were initial for the MD simulations of hydrates.

In the second step, simulations dealt with the polymers hydrated up to 60% swelling ratio. The initial hydrated systems were built with the Amorphous Cell module. To avoid long calculations, a single polymer chain occupied each unit cell. The sizes of the simulation unit cells adjusted to the final density of the system equal to 1.1 g/cm^3^ are reported in Table 1. Methods of calculation were similar to those applied in the first step, except that the electrostatic and van der Waals interactions were calculated by the 3D Ewald summation.

### 3.2. Characteristics of the Simulated System

Several functions and quantities were obtained from the simulation results. Short definitions and relationships are given below for readers’ convenience. More on this subject can be found in textbooks and monographs, e.g., in [72].

The radial distribution function (RDF) for atom pairs is defined conventionally as

(5) $$g_{AB}(r)=\rho (r)/\rho_{0},$$

where A and B denote the atoms, *ρ*(*r*) is the number density of B at a distance *r* from A, and *ρ*_0_ is the average density of B in the system.

Hydrogen bonds D–H…A in the systems were identified based on the geometric criteria: the DA distance up to 3.5 Å, the HA distance shorter than 2.5 Å, and the DHA angle deviation from the straight one up to 30° [48]. The latter criterion is of marginal importance [73].

**Figure 18:**
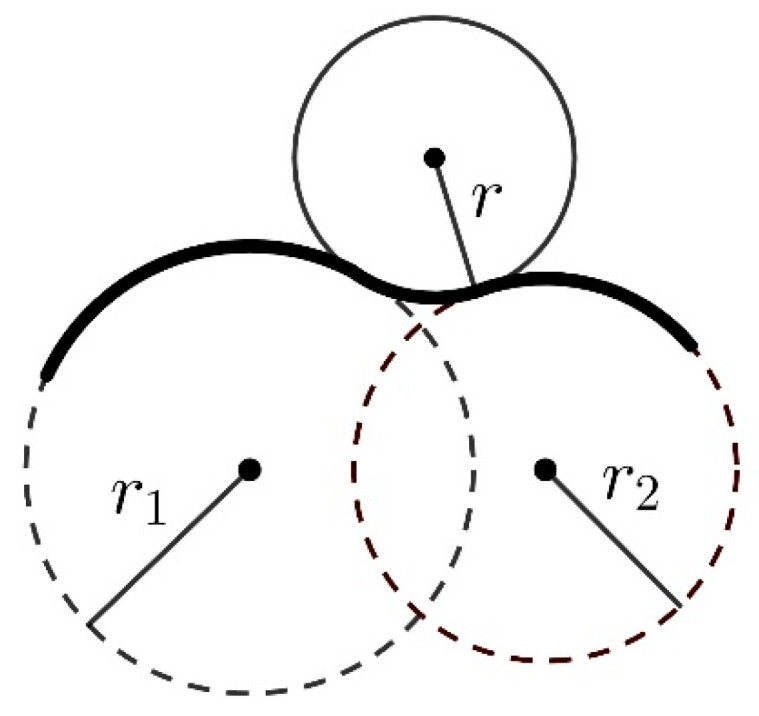
“Rolling” of a spherical probe with radius *r* over the surface of a molecule built of two atoms with van der Waals radii *r*_1_ and *r*_2_.

Free volume, *V*_f_, is operationally defined as that of the polymer material which is not occupied by the material itself but remains available for other molecules [74]. To estimate the free volumes, the Connolly algorithm was applied [75]. In this approach, the accessible molecular surface (the Connolly surface) is formed by virtually “rolling” a spherical probe over the van der Waals surface of a molecule, as illustrated in Figure 18. The free volume is outside the Connolly surface.

Fractional free volume (FFV), *f_v_*, is the ratio of the free volume *V*_f_ to the total volume of the system *V*:

(6) $$f_{v}=\frac{V_{f}}{V}.$$

The following van der Waals radii for the atoms of the polymer molecules were applied in the calculations: *r*_C_ = 1.70 Å, *r*_H_ = 1.20 Å, *r*_O_ = 1.52 Å, *r*_N_ = 1.55 Å, and *r*_P_ = 1.80 Å.

Mean square displacement (MSD), $\langle r^{2}(t)\rangle$, is defined as:

(7) $$\langle r^{2}(t)\rangle =\frac{1}{N}\sum_{i=1}^{N}{\left|\vec{r_{i}}\left(t\right)-\vec{r_{i}}\left(0\right)\right|}^{2},$$

where $\vec{r_{i}}(0)$ and $\vec{r_{i}}(t)$ are the initial and final positions of the *i*-th particle over the time interval *t*, and *N* is the number of equivalent particles.

The diffusion coefficient, *D*, is related to the MSD by Einstein’s relation:

(8) $$D=\frac{1}{6N}\underset{t\to \infty }{\lim}\frac{d}{dt}\langle r^{2}(t)\rangle .$$

The van Hove space-time correlation function, $G\left(\vec{r},t\right)$, is defined as the correlation between the particle position ${\vec{r}}^{\prime }+\vec{r}$ at the time $t^{\prime }+t$ and its position ${\vec{r}}^{\prime }$ at the time $t^{\prime }$. For a homogeneous system at equilibrium and the initial time $t^{\prime }$ = 0, the $G\left(\vec{r},t\right)$ function depends on the relative distance:

(9) $$G\left(\vec{r},t\right)=\frac{1}{N}\langle \sum_{i=1}^{N}\sum_{j=1}^{N}\delta \left(\vec{r}-\left[{\vec{r}}_{j}\left(t\right)-{\vec{r}}_{i}\left(0\right)\right]\right)\rangle$$

where the angular brackets represent the time average over the system and *δ* is the Dirac delta function. The $G\left(\vec{r},t\right)$ function can be split into the self-term $G_{s}\left(\vec{r},t\right)$ for *i* = *j* and distinct term $G_{d}\left(\vec{r},t\right)$ for *i* ≠ *j*:

(10) $$G\left(\vec{r},t\right)=G_{s}\left(\vec{r},t\right)+G_{d}\left(\vec{r},t\right),$$

where

(11) $$G_{s}\left(\vec{r},t\right)=\frac{1}{N}\langle \sum_{i=1}^{N}\delta \left(\vec{r}+{\vec{r}}_{i}\left(0\right)-{\vec{r}}_{i}\left(t\right)\right)\rangle$$

and

(12) $$G_{d}\left(\vec{r},t\right)=\frac{1}{N}\langle \sum_{i=1}^{N}\sum_{j=1,i\neq j}^{N}\delta \left(\vec{r}+{\vec{r}}_{i}\left(0\right)-{\vec{r}}_{j}\left(t\right)\right)\rangle .$$

The self-term $G_{s}\left(\vec{r},t\right)$ gives the distribution of displacements at time *t*. In this study, the $G_{s}\left(\vec{r},t\right)$ functions for water molecules in the hydrated polymers were analyzed.

## 4. Conclusions

All simulated copolymers, dry and hydrated, proved to be amorphous. The backbone chains rolled inward with outward-facing side chains. Neither the copolymer composition nor the mers arrangement affected the HEMA sidechains’ geometry. The latter remained fairly rigid even when the polymer was hydrated. Contrary to HEMA sidechains, those of MPC were flexible and coiled. That resulted in the mobility of the phosphorylcholine groups. However, the neighboring HEMA could stiffen the MPC chain. In hydrated polymers, particularly the two MPC-rich ones, water molecules penetrated voids between the MPC sidechains and uncoiled them to some extent.

Only interatomic distances suggested hydrogen bonds, as the molecules were not polarizable in these simulations. The bonds occur between the terminal hydroxyl groups of the HEMA sidechains and water molecules. The main polymer chain keeps water molecules from reaching the carbonyl oxygen atom of HEMA. Consequently, it weakens its propensity to hydrogen bonding. Nevertheless, the tendency is greater than that of the ether oxygen atoms. In this respect, carbonyl and ether groups of MPC sidechains did not differ from those of HEMA. However, the MPC proved more hydrophilic than HEMA due to the phosphate group being capable of forming hydrogen bonds through negatively charged oxygen atoms. Water molecules gathered around this and trimethylammonium groups. For the latter, this is due to electrostatic attraction only. Hydrogen bonding between water and nitrogen atoms is impossible because of the shielding methyl groups.

Non-hydrated block copolymers showed smaller free volumes than random ones. The fraction of volume available for water increased in the following order of the polymers: B13 < B37 < R13 ≈ R37. Thus, if HEMA mers separated the long MPC chains from one another, then penetration of water within the interchain spaces would be easier. Swelling of the polymers that accompanied hydration diminished the differences in the free volumes completely.

Flexible MPC side chains favored the formation of diffusion channels. A similar effect was reported for other flexible-chain polymers [57,58]. Subdiffusion of water in hydrated copolymers was evident at least up to the water content of 20% by mass. The process consisted of the “hopping” of water molecules from one “cage” in the polymer matrix to another. Between the “hops”, the molecule oscillates in the “cage” which is determined by the minimum of the potential energy of interactions. Further hydration increased the share of water resembling the bulk phase. Consequently, normal diffusion predominates in hydrated polymers with 40 and 60% of water by mass. Experimental studies confirmed such normal diffusion in polymers containing at least 30% of water [76]. Moreover, water acted as a plasticizer increasing the mobility of polymer chains.

A general agreement of the conclusions from the present simulations with experimental results reported in the literature confirmed that molecular dynamics could facilitate the selection of potential materials for SCLs.

## Figures and Tables

**Table 1:** The cell sizes for the P(MPC–*co*–HEMA) systems after 5-ns-long MD simulations.

Copolymer	Water Content by Mass (%)	Number of Water Molecules in the Cell	Unit Cell Edge (Å)
R13, B13	0	0	23.566
	10	54	24.417
	20	120	25.381
	40	321	27.943
	60	721	31.976
R37, B37	0	0	26.717
	10	78	27.673
	20	175	28.777
	40	467	31.672
	60	1050	36.248

**Table 2:** The fractional free volumes (FFVs) in P(MPC–*co*–HEMA) of different built and degrees of hydration for the probe of infinitesimal radius.

Copolymer	Fractional Free Volume (%)				
	No Water	10% H_2_O	20% H_2_O	40% H_2_O	60% H_2_O
B13	39.6	38.7	38.0	36.6	35.3
R13	39.8	38.8	38.0	36.7	35.4
B37	41.0	39.9	39.0	37.3	35.7
R37	40.8	39.9	39.0	37.2	35.8

**Table 3:** The fractional free volume (FFVs) in P(MPC–*co*–HEMA) available for water molecules, calculated with a spherical probe of a radius of 1.4 Å.

Copolymer	Fractional Free Volume (%)				
	No Water	10% H_2_O	20% H_2_O	40% H_2_O	60% H_2_O
B13	11.6	22.7	32.6	51.8	68.7
R13	15.7	23.0	33.5	51.9	68.4
B37	13.9	25.9	33.5	51.7	68.2
R37	14.6	24.0	34.5	51.7	68.1

**Table 4:** Exponents *α* in Equation (1) for MSD of water molecules in hydrated P(MPC–*co*–HEMA) copolymers.

Copolymer	*α*			
	10% H_2_O	20% H_2_O	40% H_2_O	60% H_2_O
B13	0.74	0.88	0.93	0.97
R13	0.74	0.88	0.92	0.97
B37	0.76	0.89	0.95	0.99
R37	0.79	0.90	0.95	0.99

**Table 5:** The diffusion coefficient of water in hydrated P(MPC–*co*–HEMA) copolymers.

Copolymer	*D*·10^−5^ (cm^2^ s^−1^)	
	40% H_2_O	60% H_2_O
B13	1.55	1.62
R13	1.53	1.62
B37	1.58	1.65
R37	1.58	1.65

## Data Availability

Data available from K. Filipecka-Szymczyk on personal request.
